# The insula modulates arousal-induced reluctance to try novel tastes through adrenergic transmission in the rat

**DOI:** 10.3389/fnbeh.2015.00164

**Published:** 2015-06-29

**Authors:** Sebastián Rojas, Raúl Diaz-Galarce, Juan Manuel Jerez-Baraona, Daisy Quintana-Donoso, Rodrigo Moraga-Amaro, Jimmy Stehberg

**Affiliations:** Laboratorio de Neurobiologia, Centro de Investigaciones Biomedicas, Universidad Andres BelloSantiago, Chile

**Keywords:** taste neophobia, reluctance, insular cortex, insula, adrenergic activity, arousal

## Abstract

Reluctance to try novel tastes (neophobia) can be exacerbated in arousing situations, such as when children are under social stress or in rodents, when the new taste is presented in a high arousal context (HA) compared to a low arousal context (LA). The present study aimed at determining whether adrenergic transmission at the Insula regulates the reluctance to try novel tastes induced by arousing contexts. To this end, a combination of systemic and intra-insular manipulations of adrenergic activity was performed before the novel taste (saccharin 0.1%) was presented either in LA or HA contexts in rats. Our results show that systemic adrenergic activity modulates reluctance to try novel tastes. Moreover, intra-insular microinjections of propranolol or norepinephrine (NE) were found to modulate the effects of arousing contexts on reluctance to try novel tastes. Finally, intra-insular propranolol blocked epinephrine-induced increased reluctance, while intra-insular NE blocked oral propranolol-induced decreases in reluctance and increased the reluctance to try novel tastes presented in low arousing contexts. In conclusion, our results suggest that the insula is a critical site for regulating the effects of arousal in the reluctance to try novel tastes via the adrenergic system.

## Introduction

Reluctance to novelty or Neophobia is a common adaptive behavior that ensures a cautious response to a novel stimulus until its safety has been ascertained. In animals including humans, consuming novel foods is usually accompanied by reluctance. For example, the experience of social pressure to consume novel foods in children can induce dislike for those foods (Batsell et al., 2002). The reluctance to try novel tastes was studied in the 60 s and 70 s when it was shown that domestic rats suffering from vitamin deficiency show strong decreases in their reluctance to try novel tastes, believed to ease the transition from vitamin deficient diets to novel ones (Rozin and Rodgers, 1967). Neophobia can be measured in a laboratory setting when animals (e.g., rodents) are exposed to a novel taste by itself or as a choice to water (Dunn and Everitt, 1988; Stehberg and Simon, 2011) and can be significantly increased if the novel taste is presented after stress (Dess, 1992) or in a novel context (high arousal context, HA) compared to a homecage (low arousal context, LA; De la Casa and Díaz, 2013).

This type of behavioral response has been used to measure anxiety in rodents. In fact, exposure to novel environments induces avoidance to consume novel foods (food chow rather than a particular taste dissolved in water) presented in those environments, behavior known as hyponeophagia and used to measure anxiety (Deacon, 2011).

Little is currently known about the brain areas and mechanisms that determine the reluctance to try novel tastes and how they are affected by stress and arousal. Lesion studies suggest that taste neophobia is modulated by the taste area within the Insular Cortex (or Insula, IC; Roman and Reilly, 2007; Lin et al., 2009; Stehberg et al., 2011; Moraga-Amaro et al., 2014). In the rat, the taste responsive area of the insula occupies the dysgranular and granular subregions dorsal to the rhinal fissure, from 1.5 mm posterior until 1.5 mm anterior to the Middle Cerebral artery (Paxinos and Watson, 2007), identified as taste responsive using intrinsic signal imaging (Accolla et al., 2007) as well as electrophysiologically (Yamamoto et al., 1980; Kosar et al., 1986; Ogawa et al., 1990; Bahar et al., 2004).

Although the neurotransmitters involved in the formation of novel taste memory, familiar taste memory and conditioned taste aversion have been studied in some detail (Berman et al., 2000; Bermúdez-Rattoni et al., 2004; Guzmán-Ramos et al., 2012), very few studies have attempted to study the neurotransmitters involved in taste neophobia and in arousal-induced increases in taste neophobia. This is probably due to the fact that the reluctance to try novel tastes only lasts seconds to minutes, but memories linger for days or even for a life time. Understanding the cortical mechanisms by which stress and arousal affects unconditioned spontaneous behaviors that depend directly on cortex, such as the reluctance to try novel tastes, could pave the way not only to the understanding of how stress directly affects behavior, including how it affects our perception of novelty, but may also lead to novel targets for the treatment of stress and anxiety disorders.

Early studies suggest that hyponeophagia depends on an intact brain norepinephrine system (Sahakian et al., 1983; Cole et al., 1988). During acute stress or arousal, epinephrine (EPI) is released by the adrenal medulla in response to an early sympathetic response, activating indirectly the release of brain norepinephrine (NE) from the locus coeruleus (McGaugh et al., 1996). Brain NE in turn activates the hypothalamic-adrenal axis (O’Connor et al., 2000), causes a shift from focused processing of sensory information to general scanning of the environment (Aston-Jones and Cohen, 2005; Roozendaal et al., 2009a; Sara, 2009) and enhances memory consolidation of stressful experiences (Liang et al., 1990; McGaugh and Roozendaal, 2002) in order to ensure a predictive or prompt response to a similar stressful situation in the future. NE enhancement of memory consolidation of emotionally arousing experiences is modulated by glucocorticoids (Roozendaal et al., 2009b), which have been shown to enhance memory in HA contexts or concomitant to NE administration in LA contexts (Roozendaal et al., 2006), suggesting that NE is capable of inducing high arousal experiences during low arousal situations.

Here we aim at studying the role of the adrenergic system at the insular cortex in modulating taste neophobia and arousal-induced increases in the reluctance to try novel tastes, by using a combination of systemic and intra-cortical manipulations of adrenergic activity before the presentation of saccharin as a novel taste, either at a LA (homecage) or HA context (lit novel clean cage).

## Experimental Procedures

All procedures involving animals were in accordance with the U.S. National Institutes of Health guidelines and with approval of the Bioethical Committee of the Universidad Andres Bello. Male Sprague-Dawley rats (60 days old, 200–250 g) were caged individually with free access to food and water at 22°C, under a 12-h light-dark cycle. The rats remained in their homecage throughout the study and were removed only for surgery, and briefly for drug administrations and behavioral procedures.

### Surgical Procedures

Cannulas were chronically implanted as described previously (Stehberg et al., 2012). In brief, animals were deeply anesthetized with a combination of ketamine/xylazine (0.02 μl/kg and 0.33 μl/kg, respectively), placed in a stereotaxic apparatus and their skull was surgically exposed after applying lidocaine subcutaneously into the scalp (2% HCl). Animals were then stereotaxically implanted bilaterally with a 21-gauge stainless steel guide cannulae positioned at 1.0 mm above the IC [1.2 mm anterior to Bregma, 5.4 mm lateral to the midline, and 6.7 mm ventral to the skull surface (Paxinos and Watson, 1986)]. The cannula was fixed to the skull using acrylic dental cement and secured by four screws. A stylus was placed inside the guide cannula to prevent clogging. After surgery, animals received a subcutaneous injection of ketoprofen 1% (Naxpet, laboratorio Drag pharma Chile Invetec S.A., Chile, 3 mg/Kg) and a dermal ointment consisting of a bacitracin/neomycin mixture (Laboratorio Chile, subsidiary of TEVA in Chile) was applied over the surgical area. Rats were given at least 7 days to recover from surgery before beginning experimental procedures and were handled for 10 min daily to habituate them to soft pressure on the implant throughout the recovery period to decrease microinfusion-related stress.

Intra-insular microinfusions were performed 10 min before taste presentation to avoid any behavioral effects from microinfusion discomfort. For microinfusion, the stylus was removed and a 25-gauge injection cannula was inserted through the guide cannula with its tip extending 1.0 mm beyond the guide cannula tip, into the dysgranular area of the insula, believed to be the taste responsive area according to previous literature using electrophysiology (Yamamoto et al., 1980; Kosar et al., 1986; Ogawa et al., 1990; Bahar et al., 2004) and intrinsic signal imaging (Accolla et al., 2007). Drugs were microinfused via the injection cannula, connected by PE20 tubing to Hamilton micro-syringes driven by an electronic microinfusion pump. Infusions consisted of 0.5 μl delivered at a rate of 0.25 ul/min to each hemisphere. Following drug microinfusion, the injection cannula was left in place for 3 min to allow the drug to diffuse away from the tip. Cannula placement was later determined by histology and maximal diffusion was verified by infusing 0.5 μl of India ink in a group of five rats. The maximal diffusion spread observed included the caudal insular cortex (granular, dysgranular and agranular areas) and in some cases the claustrum, capsula externa, caudoputamen and somatosensory secondary cortex.

To evaluate to which extent adrenergic activity in the insular cortex modulates arousal-induced increases in the reluctance to try novel tastes, a combination of systemic and intra-insular manipulations of adrenergic activity were performed.

### Drugs

All experimental groups were compared to a vehicle micro-infused control group. For the first experiment, intra insular propranolol (S(-)-Propranolol hydrochloride, Santa Cruz Biotechnology Inc., Dallas, TX, USA) was administered into the insular cortex at 1, 5 and 10 μg/0.5 μl dissolved in sterile saline. In the second experiment, an intra-insular microinfusion of 1 μg/0, 5 μl of NE (L-Norepinephrine hydrochloride, Sigma-aldrich, St. Louis, MO, USA) was performed. For the third experiment, systemic (oral) propranolol (Laboratorio Chile, subsidiary of TEVA, Chile) was administered dissolved in tap water for 1 h before taste presentation at a dose of 13.3 mg/kg, combined with an intra-insular microinfusion of NE at a dose of 1 μg/0.5 μl, 10 min prior to the neophobia test. For the fourth experiment, systemic epinephrine ((±) Epinephrine hydrochloride, Santa Cruz Biotechnology Inc., Dallas, TX, USA) was dissolved in sterile saline and administered i.p. 30 min before taste presentation using doses of 0.001, 0.01, 0.1 and 1 mg/kg. In the last experiment, systemic epinephrine (0.1 mg/kg) was followed by intra-insular microinjection of propranolol (1, 5 and 10 μg/0.5 μl).

### Behavioral Testing

Rats underwent water restriction during behavioral procedures. On the training phase (day 1 to day 3), animals were trained to drink from two pipettes of 10 ml each and allowed to drink their daily fluid intake within a 10 min interval per day, for three consecutive days before the test. On the experimental day animals were randomly assigned to different groups and were exposed to saccharin 0.1% as the novel taste according to the method used in Stehberg and Simon (2011). In brief, 10 min long presentations of the taste (saccharin 0.1% dissolved in water) as a choice to water (taste presentation; six pipettes containing 5 ml each alternating taste or water). Free choice tests were used and preferred over one or two bottle tests to force rats to choose and drink from at least three pipettes to meet their daily needs of fluid intake (for a discussion on the benefits of using free choice tests see (Moraga-Amaro et al., 2014). During the test, animals were offered six pipettes of 5 ml each, with a total of 30 ml so that animals are exposed to a choice of the novel taste (saccharin) and water, but are not forced to drink from either. Thus, by giving them 30 ml to choose from, the animal will not be forced to drink from all the pipettes to quench its thirst, but may instead choose to drink up to 15 ml of any of the two tastes without needing to drink more than 5 ml of the other. This way, the animal is forced to choose and to try both tastes, but not forced to drink from any particular taste. To elicit the least neophobic response, the novel taste was presented in the LA context which was the animal’s homecage. To elicit the greatest neophobic response a HA context was used, which consisted of a brightly illuminated (100 lux) clean cage void of wood shavings. Animals were put into the HA context 3 min before taste presentation and were monitored for any unusual behavior. Novel taste consumption was measured as aversion index, which represents the percentage of avoidance of the taste and is measured as the amount of water consumed divided by total liquid consumption.

### Histology

At the end of all experiments, animals were anesthetized as above and perfused intracardially with saline and 4% buffered paraformaldehyde. Brains were extracted and left afloat in 30% sucrose until its density equaled that of sucrose. The brains were sectioned in a cryostat, Nissl-stained (cresyl violet) and examined using light microscopy for cannula placement and assessment of histological lesions, defined by tissue damage and/or gliosis. Animals with injection cannula tip outside the insular cortex or showing histological lesions beyond the size of the cannula tip and guide cannula diameter were excluded from the analysis.

### Statistics

Data are expressed as mean ± standard error (SE). All experimental groups were tested for normality using the Kolmogorov-Smirnov test. Given that all data sets were found to be normally distributed, an unpaired Student’s *t* test was used when comparing two different groups and considered significant at values of *p* < 0.05. For multiple comparisons, a One-way ANOVA with Bonferroni *post hoc* tests was used, considering α significance level of *p* < 0.05.

## Results

**Figure 1:**
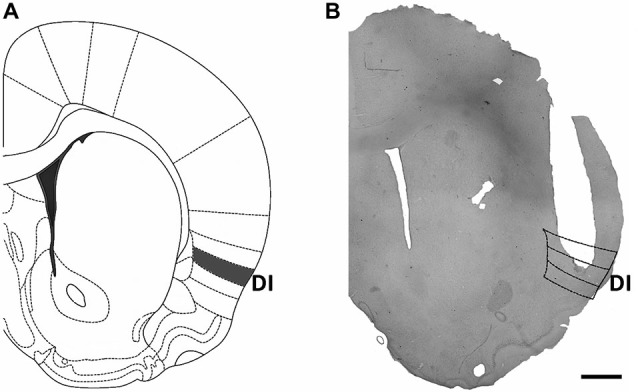
**Location of cannula implants within the insular cortex. (A)** Scheme of a brain coronal section showing the dysgranular (gustatory) area of the insular cortex, which was targeted by cannula implants. **(B)** Microphotograph of a representative cannula implant into the dysgranular (taste) area of the insular cortex. Scale bar: 1 mm.

Animals that showed a lesion larger than the size of the injection cannula or whose injection site was not located within the dysgranular or granular zones of the Insular cortex as described by Cechetto and Saper (1987) were excluded from analysis. For a scheme of the cannula locations included in the analysis see Figure 1A and for a representative microphotograph of a successful implant see Figure 1B.

**Figure 2:**
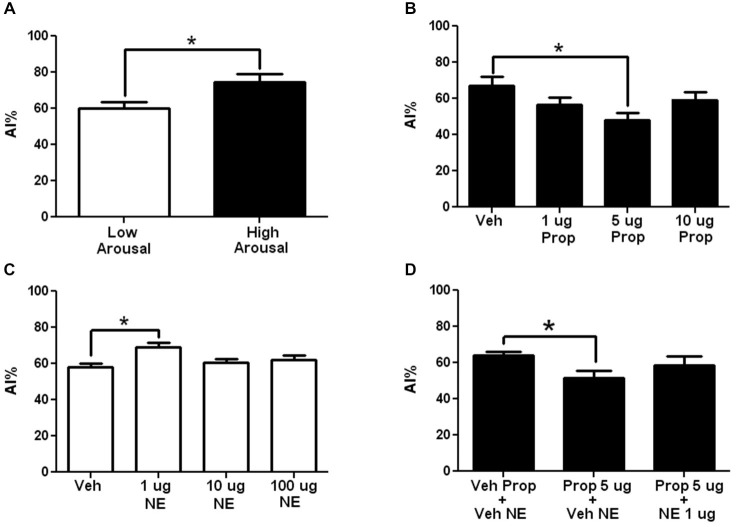
**The adrenergic antagonism by propranolol in arousal-induced taste neophobia and its modulation by the insular cortex. (A)** Comparison of the neophobic response to a new taste (saccharin 0.1%) when it is presented in a low arousal context (LA) (open bar) compared to a high arousal context (HA) (closed bar). Note that arousal induces increases in taste neophobia (*N* = 10 each, *p < 0.05). **(B)** Dose-response curve for Intra-insular propranolol in arousal-induced neophobia (taste presented in a HA context). Note that the 5 μg induced a significant decrease in arousal-induced neophobia (*N* = 12, 10, 10, 10; *p < 0.05). **(C)** Dose response curve of Intra-insular norepinephrine in arousal-induced neophobia when taste is presented in a LA context. Note that 1μg induced a significant increase in neophobia. (*N* = 15, 11, 11, 13; **p < 0.001) **(D)** Inhibition of the effects of 5 μg oral propranolol by intra-insular norepinephrine. Oral propranolol (Prop + Veh) induced a significant decrease in arousal-induced taste neophobia compared to Vehicle injected controls (Veh Prop-Veh NA), effect that was blocked when oral propranolol was followed by 5 μg of intra-insular norepinephrine (Prop + NA; *N* = 11, 10, 7; *p < 0.05; **p < 0.01; ***p < 0.001).

As can be seen in Figure 2A (open bar), presentation of the novel taste in a LA context induced reluctance to consume the novel taste (neophobia), which is reflected by a slight aversion to the taste (drinking lower amounts of the taste compared to water) reaching 60% ± 3.6 aversion, which is greater than chance drinking (50%) or preferred drinking (any value <50%). However, when the novel taste is presented in a HA context, taste aversion increases significantly to 75% ± 4.5, which reflects what is here considered as arousal-induced increase in taste neophobia (see Figure 2A, closed bar; *N* = 10 each; *p* < 0.05). To ease viewing, in all graphs except for those showing total fluid intake, experiments performed in HA contexts are shown with closed bars, while those performed in LA contexts, with open bars.

In the first experiment we aimed at testing whether adrenergic activity in the insula modulates arousal-induced increases in neophobia. For this aim propranolol was microinjected into the insular cortex prior to taste presentation in a HA context. As can be seen in Figure 2B, 5 μg of intra-insular propranolol produced a significant decrease in the reluctance to try the novel taste, suggesting that adrenergic activity at the insular cortex modulates arousal-induced increases in taste neophobia (for a curve-response graph see Figure 2B; Veh: 67 ± 5.3%, 1 μg: 56 ± 4.2% (*p* < 0.05), 5 μg: 48 ± 4.1%; 10 μg: 59 ± 4.5%; *n* = 12, 10, 10, 10 respectively).

To test if brain NE at the insular cortex can increase the reluctance to try novel tastes when the taste is presented in a LA context, in a manner similar to the arousal-induced increases in taste neophobia obtained when the taste is presented in a HA context, dose response curve of NE was microinjected into the Insular cortex, in a LA context (Exp. 2). As can be seen in Figure 2C, a significant increase in reluctance was obtained when microinjecting 1 μg of NE into the insula (for a dose-response curve see Figure 2C; veh: 58 ± 1.9%, 1 μg: 69 ± 2.6% (*p* < 0.01), 10 μg: 60 ± 1.9%, 100 μg: 62 ± 2.7%, *n* = 15, 11, 11, 13 respectively).

To test if the systemic effects of propranolol can be blocked by NE at the insular cortex, propranolol was administered orally (13.3 mg/kg) and 1 μg of NE was microinjected into the insular cortex, in a HA context. A significant decrease in neophobia was found after propranolol was administered orally (see Figure 2D, Prop + Veh NE), effect that was blocked when oral propranolol was administered together with intra-insular NE (see Figure 2D; Veh prop + Veh NE: 64 ± 2.1, Prop + Veh NE: 52 ± 3.7 (*p* < 0.05), Prop + NE: 58 ± 5.2; *N* = 11, 10 and 7 respectively).

**Figure 3:**
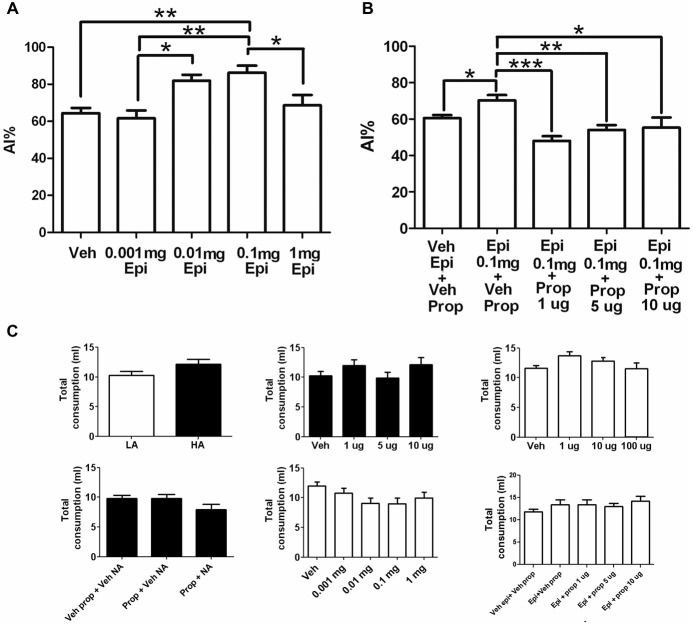
**Effects of systemic epinephrine in neophobia and its modulation by the insular cortex. (A)** Dose response curve of systemic epinephrine in a LA context. Note that 0.1 mg of epinephrine induced a significant increase in taste neophobia (*N* = 9, 10, 9, 10, 10; ***p < 0.001) when the novel taste was presented in a LA context. **(B)** Dose response of intra-insular propranolol after a systemic epinephrine when novel taste is presented in a LA context. Note that 1μg of intra-insular propranolol (Epi + Prop 1μg) blocked the increase in neophobia induced by 0.1 mg of systemic epinephrine (Epi + Veh Prop; (*N* = 8, 7, 8, 7; **p < 0.01). **(C)** Injections did not induce significant changes in total liquid consumption. Experiments in LA are shown in open bars, experiments performed in HA are shown in closed bars. Upper-middle: propranolol; left-upper right; noradrenaline; center-bottom: epinephrine. *p < 0.05; **p < 0.01; ***p < 0.001.

To test whether systemic adrenergic activity may affect taste neophobia, epinephrine was administered systemically in a LA context. Systemic administration of 0.1 mg/Kg epinephrine induced a statistically significant increase in neophobia to the taste, compared to vehicle injected controls (Figure 3A; Veh: 64 ± 2.8%, 0.001 mg/Kg: 62 ± 4.3%, 0.01 mg/Kg: 82 ± 3.3%; 0.1 mg/Kg: 86 ± 3.8% (*p* < 0.01); 1 mg/Kg: 69 ± 5.6%; *n* = 9, 10, 9, 10, 10 respectively). The 0.1 mg/kg dose had also significant differences from the 0.001 mg/Kg (*p* < 0.01) and 1 mg/Kg (*p* < 0.05) doses, while the dose of 0.01 mg/Kg showed a statistically significant difference with the 0.001 mg/kg dose (*p* < 0.05).

To determine whether the increase in reluctance to try novel tastes induced by systemic epinephrine is mediated by brain NE at the insular cortex, propranolol was microinjected into the insular cortex before taste presentation in a LA context with prior systemic injection of epinephrine. The vehicle injected group showed low neophobia as expected from a LA context (veh epi+veh prop), which was significantly increased in response to systemic epinephrine (epinephrine + Veh Prop; *p* < 0.05). All doses of intra-insular propranolol induced a significant decrease in neophobia, reaching chance drinking levels and blocking completely the effects of systemic epinephrine (for a dose-response curve see Figure 3B; Veh Epi + Veh Prop: 61 ± 1.6%, Epi + Veh Prop: 70 ± 2.9% (compared to veh; *p* < 0.05); Epi + Prop 1 μg: 48 ± 2.7% (compared to epi+veh prop; *p* < 0.001), Epi + Prop 5 μg: 54 ± 2.7% (compared to epi+veh prop; *p* < 0.01); Epi + Prop 10 μg: 55 ± 5.5% (compared to epi+veh prop; *p* < 0.05); *n* = 14, 8, 7, 8, 7 respectively). For a summary of results see Table 1.

**Table 1:** **Summary of results**.

Figure	Experiment	*N*	Groups	AI (%)	*p* value
2A	HA vs. LA	10	HA	60 ± 3.6
		10	LA	75 ± 4.5	*0.0193
2B	intra-insular Propanolol dose response curve (HA)	12	Veh	67 ± 5.3		
		10	1 μg	56 ± 4.2
		10	5 μg	48 ± 4.1	*0.0470
		10	10 μg	59 ± 4.5	
2C	Intra-insular Noradrenaline dose response curve (LA)	15	Veh	58 ± 2.6
		11	1 μg	69 ± 2.6	**0.0082
		11	10 μg	60 ± 1.9
		13	100 μg	62 ± 2.7
2D	Inhibition of oral Propanolol by intra-insular Noradrenline (HA)	11	Veh prop + Veh NA	64 ± 2.1
		10	Prop + Veh NA	52 ± 3.7	*0.0470
		7	Prop + NA	58 ± 5.2
3A	Systemic Epinephrine dose response curve (LA)	9	Veh	64 ± 2.8
		10	0.001 mg	62 ± 4.3
		9	0.01 mg	82 ± 3.3
		10	0.1 mg	86 ± 3.8	***0.0002
		10	1 mg	69 ± 5.6
3B	Epinephrine (0.1 mg) + intra-insular Propanolol dose response curve (LA)	8	Epi + Veh prop	70 ± 3.8
		7	Epi + Prop 1 μg	48 ± 2.7	**0.0022
		8	Epi + Prop 5 μg	54 ± 2.7
		7	Epi + Prop 10 μg	55 ± 5.5

Depicted from left to right are: the Figure panel where experiment is shown (Figure), a description of the experiment (Experiment), the number of animals per group (N), the groups compared (Groups), the aversion index obtained (AI%) and the P value if significant (*if p < 0.05; **if p < 0.01; ***if p < 0.001).

To ensure that the drugs used did not affect the capacity of animals to drink or their thirst, total liquid intake (water + saccharin) was measured and compared between groups, for every experiment. No significant differences were found in total liquid intake between groups in the HA vs. LA neophobia experiment (HA = 10 ± 0.7; LA = 12 ± 0.8; *p* > 0.05; Figure 3C upper left), Propranolol dose response (Veh = 10 ± 0.7; 1 μg = 12 ± 0.9; 5 μg = 10 ± 1.0; 10 μg = 12 ± 1.2; *p* > 0.05; Figure 3C upper center), the systemic Propranolol and intra-insular NE experiment (Veh Prop + Veh NE = 10 ± 0.6; Prop + Veh NE = 10 ± 0.7; Prop + NE = 8 ± 0.9, *p* > 0.05; Figure 3C lower left), or the epinephrine and intra-insular propranolol experiment (Veh Epi + Veh Prop = 12 ± 0.6; Epi + Veh Prop = 13 ± 1.0; Epi + Prop 1 μg = 13 ± 1.2; Epi + Prop 5 μg = 13 ± 0.8; Epi + Prop 10 μg = 14 ± 1.1, *p* > 0.05; Figure 3C lower right). There were non-significant differences in total fluid intake after intra-insular NE injections (Veh = 12 ± 0.4; 1 μg = 14 ± 0.7; 10 μg = 13 ± 0.6; 100 μg = 11 ± 1.1; *p* > 0.05; Figure 3C upper right) and also non-significant differences in total fluid intake after systemic epinephrine (veh = 12 ± 0.6; 0.001 mg = 11 ± 0.8; 0.01 mg = 9 ± 0.9; 0.1 mg = 9 ± 1.0; 1 mg = 10 ± 1.0; *p* > 0.05; Figure 3C lower center).

## Discussion

A summary of the experiments and results of this study can be found in Table 1. The present results suggest first, that adrenergic activity modulates the effects of arousal in taste neophobia, as systemic propranolol can reduce arousal-induced increases in neophobia (in a HA context), while epinephrine can increase neophobia in a LA context. Secondly, our results show that adrenergic activity at the insular cortex modulates arousal-induced increases in neophobia. This conclusion is supported by the fact that intra insular propranolol decreased reluctance to try novel tastes in HA contexts, while intra insular NE increased neophobia in a LA context. Both arousal-induced and epinephrine-induced increases in neophobia can be blocked by intra-insular propranolol, which may suggest that systemic adrenergic activity may be involved in arousal-induced increases in neophobia and that adrenergic activity at the insula may modulate this effect. This idea was further supported by the fact that intra-insular microinjections of NE produced an increase in taste neophobia when the taste is presented in LA contexts.

Evidence for a role of brain NE in food neophobia (or hyponeophagia) comes from studies showing that chemical NE depletion induces increased consumption of novel vs. familiar rat chow in novel contexts using 6-hydroxydopamine lesions of the noradrenergic bundle (Sahakian et al., 1983; Cole et al., 1988) or the olfactory bulb (Royet et al., 1983). Moreover, centrally acting propranolol and pindolol but not atenolol were able to decrease hyponeophagia (Shephard et al., 1982), while microinjections of NE into the basolateral amygdala induced an increase in food neophobia (Borsini and Rolls, 1984), suggesting that the Basolateral amygdala may also be involved in food neophobia. The amygdala shares several functions with the insular cortex and both interact extensively (Moraga-Amaro and Stehberg, 2012). In a study of Lin and Reilly (Lin and Reilly, 2012) using a combination of unilateral lesions of the insular cortex and amygdala, showed that Amygdala- gustatory insular cortex connections are necessary for taste neophobia.

From the present results it is possible to propose that during arousal, sympathetic activity will lead to the release of adrenal epinephrine, which in turn will trigger the release of brain NE (McGaugh et al., 1996). Brain NE will be released at the insular cortex, triggering an increase in reluctance to try novel tastes. There may be other neurotransmitters, neuromodulatory systems and brain areas involved in this process, which have not been studied in the present report.

Distinguishing a role for adrenergic activity in neophobia *per se*, from arousal-induced increases in neophobia is challenging. When a taste is presented in the LA context (namely, the animal’s homecage) the experience itself is—as the name implies—of “low arousal”, not of “no arousal”. This means that the decrease in neophobia induced by propranolol when the taste is presented in a LA context may reflect a role for the adrenergic system in neophobia *per se*, but may also reflect an effect of propranolol on the arousal—despite being low—that the presentation of the novel taste may induce.

In a study published by Roozendaal and Cools (1994), Wistar rats were placed in an openfield and divided according to their exploratory behavior (less or more than 8 min to habituate and 48 meters locomotion per 30 min) into high and low responders. Then they were microinjected into the basolateral amygdala with either beta-adrenergic antagonist propranolol or beta-adrenergic agonist isoproterenol and 5 min later presented with a choice of novel and familiar chow in a HA context. Interestingly, propranolol reduced food neophobia only in the high responders, while isoproterenol decreased food neophobia only in low responders (Roozendaal and Cools, 1994). This suggests that the effects of adrenergic manipulations may depend on the animals’ responsiveness to stress or basal levels of anxiety. It remains to be determined whether this holds true for the insula adrenergic manipulations, or it may be unique to amygdala. If so, it may help distinguishing a role for the amygdala associating the taste response with the basal level of anxiety of each animal. In the present study no screening tests were performed to distinguish responders. More studies are required to assess this issue.

As can be seen in Figure 3C, the different injections did not interfere with the animals’ capacity to drink or their thirst. This is important, as is widely known that exposure to novel contexts induces anxiety, while adrenergic activity also mediates anxiety (Nesse et al., 1984). Thus, here we show that the change in anxiety by contexts or drugs used affected the rats’ preference for the novel taste, but not their capacity to drink or their overall thirst.

In conclusion, here we show that the adrenergic system modulates the effects of arousing contexts in the reluctance to try novel tastes via the insular cortex, suggesting that the insula may be a cortical site critical for modulating the effects of arousal in gustatory behavior. Given that hyponeophagia is used as a measure of anxiety, further studies are required to determine whether the role of the insular cortex may go beyond taste reluctance and modulate stress and anxiety.

## Conflict of Interest Statement

The authors declare that the research was conducted in the absence of any commercial or financial relationships that could be construed as a potential conflict of interest.
